# The Use of Mental Health Services by Dually-Eligible Nursing Home Residents

**DOI:** 10.1093/geroni/igaf122.401

**Published:** 2025-12-31

**Authors:** Howard Degenholtz, Michael Sharbaugh, Lingshu Xue, Paige Semple, Jie Li, Keri Kastner

**Affiliations:** University of Pittsburgh, Pittsburgh, Pennsylvania, United States; University of Pittsburgh, Pittsburgh, Pennsylvania, United States; University of Pittsburgh, Pittsburgh, Pennsylvania, United States; University of Pittsburgh, Pittsburgh, Pennsylvania, United States; University of Pittsburgh, Pittsburgh, Pennsylvania, United States; University of Pittsburgh, Pittsburgh, Pennsylvania, United States

## Abstract

Previous research on mental health service among nursing home residents has documented high rates of psychoactive medication use and increasing prevalence of people with severe mental illness (SMI). This is the first study to examine how the delivery of mental health services has changed to meet the needs of this population. We merged Medicaid and Medicare claims for mental health services visits for dually-eligible nursing home residents in Pennsylvania from 2016 to 2022 provided by mental health specialists (e.g., psychiatrists, psychologists, social workers) and non-specialists (e.g., internal medicine, family medicine, advance practitioners) with nursing home minimum data set assessments. From 2016 to 2022, the total census declined by 24% (67,229 to 51,370) residents, however the dual-eligible population dropped by 46% (45,164 to 24,291). While the prevalence of schizophrenia, bipolar disorder and depression increased, Alzheimer’s Disease and Related Dementia declined. The proportion of residents with any mental health visit increased from 25% to 37%, mainly due to doubling of visits by non-mental health specialists (from 6% to 14%). Mental health visits increased faster in nursing homes with a low prevalence of SMI (< 8%), from 15.09% to 30.49% compared to nursing homes with high prevalence of SMI ( > =21%) from 16% to 15%. There has been a long-term shift in the type of people living in US nursing homes. We found important shifts in process of care for this population that raise important questions about the quality of mental health care provided by non-specialists in the nursing home setting.

